# Development of a Hypertension Electronic Phenotype for Chronic Disease Surveillance in Electronic Health Records: Key Analytic Decisions and Their Effects

**DOI:** 10.5888/pcd20.230026

**Published:** 2023-09-14

**Authors:** Katherine H. Hohman, Bob Zambarano, Michael Klompas, Hilary K. Wall, Emily M. Kraus, Thomas W. Carton, Sandra L. Jackson

**Affiliations:** 1 National Association of Chronic Disease Directors, Decatur, Georgia; 2 Commonwealth Informatics, Waltham, Massachusetts; 3 Department of Population Medicine, Harvard Medical School and Harvard Pilgrim Health Care Institute, Boston, Massachusetts; 4 Division for Heart Disease and Stroke Prevention, National Center for Chronic Disease Prevention and Health Promotion, Centers for Disease Control and Prevention, Atlanta, Georgia; 5 Independent Consultant to Public Health Informatics Institute, a program of the Task Force for Global Health, Decatur, Georgia; 6 Louisiana Public Health Institute, New Orleans, Louisiana

## Abstract

**Introduction:**

Modernizing chronic disease surveillance with electronic health record (EHR) data may provide better data to improve hypertension prevention and control, but no consensus exists for an EHR-based surveillance definition for hypertension. The Multi-State EHR-Based Network for Disease Surveillance (MENDS) pilot surveillance system was used to develop and test an electronic phenotype for hypertension.

**Methods:**

We used MENDS data from 1,671,544 patients in Louisiana to examine the effect of different analytic decisions on estimates of hypertension prevalence. Decisions included 1) whether to restrict surveillance to patients with recent blood pressure measurements, 2) varying the number and recency of encounters to define the population at risk of hypertension, 3) how to define hypertension (diagnosis codes, antihypertensive medication, blood pressure measurements, or combinations of these), and 4) how to handle multiple blood pressure measurements on the same day. Results were compared with independent estimates of hypertension prevalence in Louisiana from the Behavioral Risk Factor Surveillance System (BRFSS).

**Results:**

Applying varying criteria resulted in hypertension prevalence estimates ranging from 19.7% to 59.3%. A hypertension surveillance strategy that includes a population with at least 1 clinical encounter with measured blood pressure in the previous 2 years and identifies hypertension using all available data (≥1 diagnosis code, ≥1 antihypertensive medication, and ≥2 elevated blood pressure values ≥140/90 mm Hg on separate days) generated estimates in line with population-based survey data. This definition estimated the crude 2019 hypertension prevalence in the state of Louisiana as 43.4% (age-adjusted, 41.0%), comparable with the crude BRFSS estimate of 39.7% (age adjusted, 37.1%).

**Conclusion:**

Applying different criteria to define hypertension using EHR data has a large effect on hypertension prevalence estimates. The proposed electronic phenotype generates hypertension prevalence estimates that align with independent estimates from BRFSS.

**Summary**

**What is already known on this topic?**

Previous studies have identified hypertension from electronic health records (EHRs), but the effects of analytic decisions on prevalence estimates have not been characterized, and a consensus definition has not been established.

**What is added by this report?**

This report addresses a gap in the literature by providing results and analytic interpretations related to different decision points in the EHR-based electronic phenotype development process and proposes an optimal definition for EHR-based hypertension.

**What are the implications for public health practice?**

Analytic decisions have a large effect on EHR-based estimates of hypertension prevalence. Parties working to advance chronic disease data modernization using EHR data can apply this EHR-based definition for surveillance of hypertension prevalence and control.

## Introduction

Electronic health record (EHR) systems provide an opportunity to use timely and detailed clinical data for public health surveillance, especially to monitor chronic conditions for which up-to-date surveillance information is sparse (1). Enhanced chronic disease surveillance tools can advance public health efforts to improve chronic disease prevention and control by helping to identify populations at greatest risk of disease; monitoring populations and subpopulation trends; and measuring the design, placement, and effect of public health interventions in a timely and efficient manner. Hypertension prevalence and control are surveillance priorities for which repeated vital measurements, such as blood pressure measurements, are captured in the EHR and can be used to identify cases and assess hypertension control. Surveillance of hypertension is a priority because this chronic condition increases the risk for heart disease and stroke — 2 of the top 5 leading causes of death in the US — and its prevention and control can result in large health benefits, such as reduced risk of stroke, heart disease, and end-stage renal disease (2,3).

Various analyses to define hypertension using data from EHRs have been attempted (4–9), but detailed information is lacking on analytic decisions in the development of electronic phenotypes (e-phenotypes) to identify hypertension cases and assess control. Gaps in the literature include how best to define hypertension (diagnosis codes, prescriptions for antihypertensive medications, elevated high blood pressure measures, or a combination of these); how to handle patients who have multiple, potentially conflicting blood pressure measures in a single day; whether to limit the surveillance population to patients who have measured blood pressure results documented in the EHR; and whether to apply a minimum number and recency of encounters to include patients in surveillance estimates. Often, a starting point for e-phenotype development is adopting or adapting established electronic clinical quality measures (eCQMs) (10). These measures aim to quantify and track the quality of health care services but may not cover conditions of importance to public health surveillance or may not be defined optimally for public health use. Although an eCQM exists for controlling high blood pressure, no eCQM exists for hypertension prevalence, a condition of public health importance.

Tests of different definitions applied to a large EHR data population and their effect on hypertension estimates have not been reported. The objective of this study was to address this information gap by describing the analytic decisions to refine a hypertension e-phenotype and the effect of these decisions on estimates of hypertension prevalence and control. We used the Behavioral Risk Factor Surveillance System’s (BRFSS’s) independent estimates of hypertension prevalence in a single US jurisdiction as a comparison standard against which to assess the effect of analytic decisions.

## Methods

We analyzed data from the Multi-State EHR-Based Network for Disease Surveillance (MENDS) pilot chronic disease surveillance system (11). MENDS is a data modernization demonstration project funded by the Centers for Disease Control and Prevention (CDC). Because it is public health surveillance, MENDS is exempt from institutional review board review. MENDS aims to leverage EHR data from 5 large partner networks across the US to generate timely estimates of chronic disease prevalence and management. MENDS is designed to allow public health agencies to monitor trends to inform policies, plan programs, and evaluate public health initiatives. MENDS is modeled on and leverages software from MDPHnet — a distributed network for chronic disease surveillance in Massachusetts (12). In addition to using the 3 open-source software platforms from MDPHnet — Electronic medical record Support for Public health (ESP) (13), PopMedNet (14), and RiskScape (15) — MENDS also adopted MDPHnet’s existing chronic disease algorithm (16). MENDS uses a multistage approach to data quality and validation that includes 1) testing to ensure the system is storing a valid representation of the source data, 2) characterizing the data to ensure they provide valid indicators of clinically meaningful population parameters for epidemiology, 3) validating internal algorithms to ensure the code is correctly identifying conditions as specified, and 4) validating externally to ensure valid estimates of the conditions being studied. In spring 2022, the MENDS validation team explored the effects of different analytic decisions on estimates of hypertension prevalence and control in a multistate EHR-based surveillance system. We examined each decision point for clinical face validity, data quality improvement, and effect on hypertension estimates relative to BRFSS estimates.

The study population for this exploration was Research Action for Health Network (REACHnet) (17), operated by the Louisiana Public Health Institute, 1 of the 5 data contributors participating in MENDS. Data contributors provide data to MENDS on behalf of multiple data owners (eg, health systems) and partner with data users from proximal state or local health departments. The total number of patients in MENDS contributed by REACHnet was more than 5 million as of May 2022. REACHnet has 4 clinical partners in Louisiana and Texas and uses privacy-preserving record linkage to link data across sites. We used data on the adult (aged ≥20 y) patient population in Louisiana (N = 1,671,544) in REACHnet (Table 1) to explore the following key decision points in 2019, the last full calendar year before the health care effects of the COVID-19 pandemic:

**Table 1:** Demographic Characteristics of Adult Population (Aged ≥20 y) in Louisiana, REACHnet, 2019^a^

Characteristic	Louisiana REACHnet, no. (%) (N = 1,671,544)
**Sex**	
Female	935,112 (55.9)
Male	735,978 (44.0)
Unknown	454 (<0.1)
**Age group, y**	
20-24	141,508 (8.5)
25-34	320,180 (19.2)
35-44	296,768 (17.8)
45-54	271,043 (16.2)
55-64	305,090 (18.3)
≥65	336,955 (20.2)
**Race**	
American Indian or Alaska Native	5,840 (0.3)
Asian	25,740 (1.5)
Black or African American	545,287 (32.6)
Native Hawaiian or Other Pacific Islander	549 (<0.1)
White	976,882 (58.4)
Multiple race	149 (<0.1)
Other race	56,407 (3.4)
Unknown	60,690 (3.6)
**Ethnicity**	
Hispanic	92,640 (5.5)
Not Hispanic	149,7826 (89.6)
Unknown	81,078 (4.9)

a Data source: Research Action for Health Network (REACHnet) (17), operated by the Louisiana Public Health Institute, 1 of the 5 data contributors participating in the Multi-State EHR-Based Network for Disease Surveillance (MENDS) pilot chronic disease surveillance system (11).

1. Should the surveillance population of interest include all patients who have had any health care encounter or just those with at least 1 measured blood pressure?
2. Should the surveillance population be limited to patients with encounters within the preceding 1 year or 2 years? In addition, should the population be limited to patients with a minimum of 1 encounter or 2 encounters within the surveillance period?
3. Should patients with hypertension be identified by using diagnosis codes, medication potentially prescribed for hypertension (antihypertensive medication), elevated blood pressure values, or a combination of these criteria?
4. When multiple blood pressure values are available for a single patient on a single day, which values should be used?

In general, the availability of REACHnet data has a 6-month lag. Had the COVID-19 pandemic not caused us to focus our analysis (conducted in spring 2022) on data from 2019, we would have been able to use data current as of November 2021. The comparison reference for hypertension prevalence was the crude and age-adjusted BRFSS estimate for Louisiana in 2019. We used the BRFSS measure for hypertension *awareness* — adults who have been told they have high blood pressure (18). We selected Louisiana for this comparison because REACHnet’s largest data coverage is in Louisiana, where it covers 31% of the state population (19). BRFSS does not have estimates for hypertension *control*, so we did not make this comparison.

### Surveillance population

Individuals interact with the health care system for various reasons, such as illness, injury, prevention, or prescription refills. Not all health care interactions result in the collection of information needed to routinely assess, diagnose, and treat hypertension, and not all health care providers are accounted for in a given system. For example, data on some patients might be captured in one system when they visit a specialist. Some clinicians, particularly specialists, defer to primary care physicians or cardiologists for the management of a patient’s blood pressure and, therefore, do not measure blood pressure or reliably record antihypertensive medication. These primary care physicians may not be part of a participating data network. Thus, a proportion of patients may be in a system whose records do not contain the minimum data needed for valid hypertension assessment. Conversely, if the strategy for identifying hypertension includes diagnosis codes or medications, then excluding patients without measured blood pressures may unnecessarily limit the size of the surveillance population or bias the surveillance population toward patients who are more engaged or under more intensive care.

We explored 5 scenarios to define the population for estimating the prevalence of hypertension: 1) a patient population with at least 1 clinical encounter in 2018 or 2019 (Denominator 1), 2) a patient population with at least 1 clinical encounter with measured blood pressure in 2018 or 2019 (Denominator 2), 3) a patient population with at least 1 clinical encounter with measured blood pressure in 2019 (Denominator 3), 4) a patient population with at least 2 clinical encounters with measured blood pressure in 2018 or 2019 (Denominator 4), and 5) a patient population with at least 2 clinical encounters with measured blood pressure in 2019 (Denominator 5). The number of people in the surveillance population is the denominator for the hypertension prevalence measure.

### Hypertension case criteria

EHR data provide multiple data elements, such as diagnosis codes, medication prescriptions, and blood pressure values, that may be used for hypertension case identification. Pertinent diagnosis codes for hypertension include *International Classification of Diseases, Ninth Revision* (ICD-9-CM) 401.x essential hypertension, ICD-9 405.x secondary hypertension (20), *International Classification of Diseases, Tenth Revision* (ICD-10-CM) I10 essential hypertension, and ICD-10-CM I15 secondary hypertension (21). Antihypertensive medication includes selected diuretics, calcium channel antagonists, angiotensin-converting enzyme inhibitors, angiotensin receptor blockers, β-blockers, and α antagonists (Box). Pertinent blood pressure values include elevated systolic measures of ≥140 mm Hg or elevated diastolic measures of ≥90 mm Hg on at least 2 separate days. We explored each of these criteria independently and in combination to identify people with hypertension. The number of people identified as hypertension cases is the numerator for the hypertension prevalence measure. We used only outpatient data.

**Box. Generic Drug Names of All Antihypertensive Medications Included by Study Team for Surveillance of Electronic Health Records**

**Table:** 

Antihypertensive medications				
acebutolol	chlorthalidone	guanfacine	metoprolol	prazosin
aliskiren	clevidipine	hydralazine	moexipril	propranolol
amlodipine	clonidine	hydrochlorothiazide	nadolol	quinapril
atenolol	diltiazem	indapamide	nebivolol	ramipril
benazepril	doxazosin	irbesartan	nicardipine	spironolactone
betaxolol	enalapril	isradipine	nifedipine	telmisartan
bisoprolol	eplerenone	labetalol	nisoldipine	terazosin
candesartan	eprosartan	lisinopril	olmesartan	trandolapril
captopril	felodipine	losartan	perindopril	valsartan
carvedilol	fosinopril	methyldopa	pindolol	verapamil

### Multiple blood pressure readings on a single day

When individuals interact with the health care system, multiple blood pressure values are sometimes recorded on a single day. There may be multiple measurements recorded during 1 encounter (for example, if a clinician suspects an initial measure may be an outlier), accidental duplicate entries, or multiple encounters in 1 day, each with its own blood pressure value. These multiple values may affect hypertension estimates. Many methods exist for treating multiple blood pressure values taken on a single day to assess hypertension prevalence and control. We explored the following 5 approaches, some drawn from the literature and others aligned with existing methodologies:

1. Mean arterial pressure (MAP) (22): For each blood pressure value, the MAP is calculated by using the equation [(2 × diastolic blood pressure) + systolic blood pressure]/3. We used the blood pressure that corresponded to the lowest MAP as the reading for the day. This is a common strategy used by intensive care physicians for integrating systolic and diastolic readings into a single metric to guide treatment and facilitate comparisons over time.
2. Average readings: The average of all systolic blood pressure values and the average of all diastolic blood pressure values were used as the reading for the day. This approach decouples paired systolic and diastolic values but is conceptually simple and mirrors the approach used by the National Health and Nutrition Examination Survey (NHANES) (23).
3. Lowest systolic blood pressure and lowest diastolic blood pressure: The lowest systolic and lowest diastolic values were used as the reading for the day. This approach was informed by the Controlling High Blood Pressure eCQM (24). This approach also decouples paired systolic and diastolic values.
4. Lowest systolic and accompanying diastolic blood pressure.
5. Lowest diastolic and accompanying systolic blood pressure.

To avoid overinflating the hypertension estimate, we used a conservative approach — taking the lowest blood pressure values (vs highest) in approaches 3, 4, and 5. For the assessment of elevated blood pressure values and hypertension control, we explored each method for determining which blood pressure to use on a given day when multiple measures for the day existed.

### Controlled hypertension

Hypertension control status for patients with hypertension was assigned according to the patient’s most recent blood pressure value starting with the day of diagnosis. We excluded from assessment of control patients lacking diagnosis codes for hypertension (ICD-9 401.x essential hypertension, or ICD-10 I10 essential hypertension).

## Results

### Surveillance population

Varying the requirements for blood pressure measurement affected the size of the denominator (the surveillance population) and the corresponding prevalence of hypertension (Table 2). If the criterion of having at least 1 clinical encounter with measured blood pressure in 2018 or 2019 was implemented, the denominator of patients was reduced by 57% (from 1,368,049 to 586,652) compared with the denominator of patients with at least 1 clinical encounter in 2018 or 2019 (regardless of whether blood pressure was measured). Of those excluded when the blood pressure criterion was added, 0.7% had a hypertension diagnosis and 1.2% had prescriptions for antihypertensive medication. The denominator was further reduced when the surveillance time span was shortened from 2 years to 1 year (from 586,652 to 477,381 — a 19% decrease) or when at least 2 blood pressure values were required instead of 1 blood pressure value in 2018 or 2019 (from 586,652 to 435,870 — a 27% decrease).

**Table 2:** Effect of Changing the Surveillance Denominator Relative to Criteria for Hypertension Case Identification 2019^a^

Criteria	Denominator 1: ≥1 clinical encounter in 2018 or 2019 (N = 1,368,049)	Denominator 2: ≥1 clinical encounter with measured blood pressure in 2018 or 2019 (N = 586,652)^b^	Denominator 3: ≥1 clinical encounter with measured blood pressure in 2019 (N = 477,381)	Denominator 4: ≥2 clinical encounters with measured blood pressure in 2018 or 2019 (N = 435,870)	Denominator 5: ≥2 clinical encounters with measured blood pressure in 2019 (N = 328,165)
Any hypertension diagnosis	201,551 (14.7)	195,914 (33.4)	179,168 (37.5)	178,895 (41.0)	151,309 (46.1)
Any ≥2 elevated blood pressure measurements^c^	142,496 (10.4)	141,941 (24.2)	131,618 (27.6)	141,085 (32.4)	122,012 (37.2)
Any antihypertensive medication	139,989 (10.2)	130,675 (22.3)	122,151 (25.6)	122,216 (28.0)	106,278 (32.4)
Any hypertension diagnosis OR any ≥2 elevated blood pressure^c^ measurements	247,908 (18.1)	241,807 (41.2)	219,411 (46.0)	224,472 (51.5)	186,906 (57.0)
Any hypertension diagnosis OR any antihypertensive medication	228,027 (16.7)	213,908 (36.5)	194,504 (40.7)	194,026 (44.5)	163,256 (49.7)
Any hypertension diagnosis OR antihypertensive medication OR ≥2 elevated blood pressure measurements^c^	269,216 (19.7)	254,661 (43.4)	230,222 (48.2)	234,492 (53.8)	194,670 (59.3)

a Data source: Research Action for Health Network (REACHnet) (17), operated by the Louisiana Public Health Institute, 1 of the 5 data contributors participating in the Multi-State EHR-Based Network for Disease Surveillance (MENDS) pilot chronic disease surveillance system (11). All values are number (percentage).

b The algorithm that generates hypertension prevalence estimates that align with clinical reasoning and expected levels of hypertension prevalence compared with available surveillance estimates.

c ≥2 Elevated blood pressure values (140/90 mm Hg) on ≥2 separate occasions in a single year.

Hypertension prevalence estimates were dependent on the strategy used to define the surveillance population (ie, the denominator). For example, when we restricted the denominator to those with at least 1 clinical encounter with measured blood pressure in 2018 or 2019, compared with having at least 1 clinical encounter regardless of whether blood pressure was measured, hypertension prevalence (determined by diagnosis, antihypertensive medication, or ≥2 elevated blood pressures) increased from 19.7% to 43.4% (Table 2). We selected a denominator requiring at least 1 clinical encounter with measured blood pressure in 2018 or 2019 (Denominator 2) for subsequent analyses because it was the least restrictive denominator that still allowed for ample opportunity for hypertension assessment (ie, captured ≥1 blood pressure value, indicating a potential primary care visit).

### Hypertension prevalence

When we used Denominator 2 as the basis for analysis, and the combination of all 3 criteria for hypertension case identification (diagnosis codes, prescription for antihypertensive medication, or ≥2 elevated blood pressures) to calculate the numerator of patients with hypertension, the crude hypertension prevalence in 2019 for this population was 43.4% (age-adjusted, 41.0%) (Table 3). This estimate was comparable with the 2019 BRFSS crude estimate of 39.7% of adults in Louisiana who recall having been told they had high blood pressure (age-adjusted, 37.1%) (Table 3). When we examined each criterion for hypertension case identification individually, diagnosis codes yielded a hypertension prevalence of 33.4%; high blood pressures yielded a hypertension prevalence of 24.2%, and antihypertensive medication yielded a hypertension prevalence of 22.3%. When we combined 2 criteria, the pairing of diagnosis codes with elevated blood pressures yielded a hypertension prevalence of 41.2%, and the pairing of diagnosis code with antihypertensive medication yielded a prevalence of 36.5%. Overall, 77% of potential hypertension cases had a diagnosis code for hypertension (Figure).

**Table 3:** Comparison of MENDS Hypertension Prevalence Estimates in Louisiana^a^ With Other Available Public Health Estimates

Source	Measure detail	Hypertension prevalence estimate, %		Description
		Crude	Age-adjusted	
National Health and Nutrition Examination Survey (NHANES) (25,26)	National (2017–2018): SBP ≥130 mm Hg or DBP ≥80 mm Hg	48.2	45.4	NHANES hypertension case definition blood pressure ≥130 mm Hg systolic and/or ≥80 mm Hg diastolic and/or currently taking antihypertensive medications
	National (2017–2020): SBP ≥140 mm Hg or DBP ≥90 mm Hg	Unpublished	32.9	NHANES case definition blood pressure ≥140 mm Hg systolic and/or ≥90 mm Hg diastolic and/or currently taking antihypertensive medications
Behavioral Risk Factor Surveillance System (BRFSS) (18)	National (2019) median of 50 state estimates	32.3	Unpublished	BRFSS hypertension case definition is based on self-report of “Have you ever been told by a provider that you have high blood pressure?”
	Louisiana (2019)	39.7	37.1	
Multi-State EHR-Based Network for Disease Surveillance (MENDS)	Louisiana (2019): SBP ≥140 mm Hg or DBP ≥90 mm Hg	43.4	41.0	MENDS hypertension case definition uses a combination of ≥1 diagnosis code, 2 elevated blood pressure readings (SBP ≥140 mm Hg or DBP ≥90 mm Hg), or antihypertensive medication and a denominator population of patients with ≥1 blood pressure measurement in the preceding 2 years

Abbreviations: DBP, diastolic blood pressure; SBP, systolic blood pressure.

a Data source: Research Action for Health Network (REACHnet) (17), operated by the Louisiana Public Health Institute, 1 of the 5 data contributors participating in the Multi-State EHR-Based Network for Disease Surveillance (MENDS) pilot chronic disease surveillance system (11).

**Figure:**
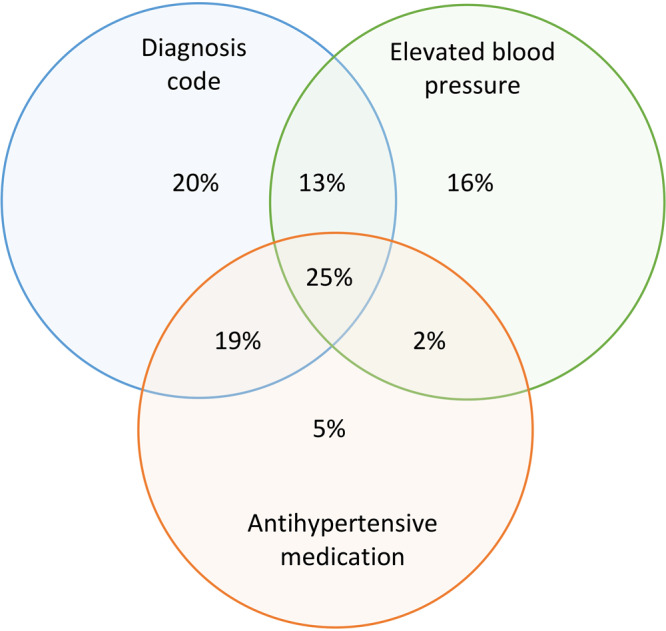
Contribution of 3 criteria for hypertension case definition to an estimated overall hypertension prevalence of 43.4% in Louisiana, 2019. The denominator for estimating prevalence was the population of patients with ≥1 blood pressure measurement in 2018 or 2019 and 3 criteria for hypertension case identification (a combination of ≥1 diagnosis code, 2 elevated blood pressures, and antihypertensive medication). Data source: Research Action for Health Network (REACHnet) (17), operated by the Louisiana Public Health Institute, 1 of the 5 data contributors participating in the Multi-State EHR-Based Network for Disease Surveillance (MENDS) pilot chronic disease surveillance system (11).

**Table:** 

Criteria	Percentage
Diagnosis code only	20
Elevated blood pressure only	16
Antihypertensive medication only	5
Diagnosis code and elevated blood pressure only	13
Diagnosis code and antihypertensive medication only	19
Elevated blood pressure and antihypertensive medication only	2
Diagnosis code, elevated blood pressure, and antihypertensive medication	25

### Multiple blood pressure readings on a single day

The 5 methods used to address multiple blood pressure values in a single day did not meaningfully affect hypertension prevalence or control; results differed by less than a single percentage point (Table 4). Hypertension prevalence ranged from 43.4% to 43.8%, depending on the method used. Hypertension control ranged from 67.1% to 67.5%, depending on the method used. About one-fifth of the population (19%) had a day with multiple blood pressure values. Eight percent (297,991 of 3,760,807) of days in 2018 and 2019 with a blood pressure value had multiple blood pressure values.

**Table 4:** Hypertension Prevalence and Control Based on Different Methods of Handling Multiple Blood Pressure Values on the Same Day^a^

Method	Hypertension prevalence, %	Hypertension control, %
Mean arterial pressure	43.7	67.4
Average of blood pressure measurements^b^	43.4	67.1
Lowest systolic and diastolic blood pressure	43.7	67.5
Minimum systolic blood pressure	43.7	67.5
Minimum diastolic blood pressure	43.8	67.3

a Data source: Research Action for Health Network (REACHnet) (17), operated by the Louisiana Public Health Institute, 1 of the 5 data contributors participating in the Multi-State EHR-Based Network for Disease Surveillance (MENDS) pilot chronic disease surveillance system (11).

b Average of blood pressures takes the average of all systolic blood pressures and the average of all diastolic blood pressures as the value for the day. Results use a denominator population of patients with ≥1 blood pressure measurement in the preceding 2 years and a 3-criterion electronic phenotype for hypertension case identification (a combination of ≥1 diagnosis code, 2 elevated blood pressure readings, or antihypertensive medications).

## Discussion

Tracking hypertension prevalence and control trends, characterizing affected populations, and assessing the effect of prevention and management programs are public health priorities that can be supported by using clinical data. Automated analyses of EHR data drawn from multiple large practices covering prioritized jurisdictions could facilitate accurate, timely, and clinically detailed surveillance for hypertension prevalence and control. Critical to this process is the creation of a rigorous and credible surveillance definition for identifying hypertension. Such a surveillance definition requires a series of analytic decisions, including how best to state the denominator population at risk, which electronic criteria to use to define hypertension cases as the numerator, and how to handle patients who have multiple potentially conflicting blood pressure values on a given day. The selected MENDS algorithm leveraged a denominator population of patients with at least 1 blood pressure measurement in the preceding 2 years, used a 3-criterion e-phenotype for hypertension case identification (a combination of ≥1 diagnosis code, 2 elevated blood pressure readings, and a prescription for ≥1 antihypertensive medication), and averaged blood pressure measurements when a patient had multiple measures on a single day.

The results of each scenario — defining the surveillance population of interest, selecting appropriate criteria for case identification, and determining how to incorporate multiple blood pressures in a single day — affected the size of the surveillance population and estimates of hypertension prevalence and control, sometimes to a large extent. For example, adding a requirement for at least 1 measured blood pressure in the preceding 2 years dropped the size of the denominator population by more than half, compared with including all patients with encounters in the preceding 2 years (1.4 million vs 0.6 million). This reduction in the denominator population was associated with a doubling of the estimated prevalence of hypertension regardless of the strategy for defining the numerator (diagnosis codes, antihypertensive medications, measured high blood pressures, or a combination of these). Inclusion of the requirement for at least 1 measured blood pressure narrowed the breadth of health care encounters included in the estimate to those that provide adequate opportunity for hypertension assessment (eg, a primary care visit rather than a mental health encounter), but it may also exclude some encounters in which a blood pressure measurement would be expected but did not happen. This result mirrors the work of Cocoros et al, who demonstrated the large effect of varying the denominator definition on disease prevalence estimates using EHR data (27). Changing the denominator population to use the criterion of having at least 1 encounter with measured blood pressure in the preceding 2 years brought the MENDS hypertension prevalence measure in line with the BRFSS hypertension prevalence estimate for Louisiana (crude, 43.4% vs 39.7%; age-adjusted, 41.0% vs 37.1%). One might expect a slightly higher estimate in MENDS given that a health care population is seeking care and potentially should have a higher hypertension estimate than the underlying general population. MENDS estimates of hypertension may also be greater than BRFSS estimates because the MENDS algorithm could be capturing data on those who have hypertension based on measured blood pressure but who are undiagnosed, or “hiding in plain sight” (28), a population not likely captured in BRFSS. To define the surveillance population for whom to assess hypertension — given that for patients with no blood pressure measures, the opportunity to assess, identify, treat, and manage hypertension was lacking — we opted to define the denominator population to assess hypertension prevalence and control as individuals who had a least 1 encounter with measured blood pressure in the preceding 2 years.

Changing the strategy for identifying hypertension cases was also associated with a nearly 2-fold change in the estimated prevalence of hypertension. Defining hypertension on the basis of prescriptions for antihypertensive medication alone, for example, led to an estimated hypertension prevalence of 22.3%, whereas also considering diagnosis codes and elevated blood pressure measures led to an estimated hypertension prevalence of 43.4%. However, importantly, estimates of hypertension prevalence using diagnosis codes alone missed approximately one-quarter of cases that were detected when all 3 criteria were considered. Therefore, we opted to use all 3 criteria to identify a hypertension case (diagnosis codes, antihypertensive medication, and ≥2 elevated blood pressures within a year). This 3-criterion definition differed from the e-phenotype selected by Peng et al, which used a combination of diagnosis codes and blood pressure values but did not include medication (8). In MENDS, the use of antihypertensive medications contributed to 5% of the cases identified. The difference between selected e-phenotypes may be due to differences in study population (United Kingdom vs US) or data source (sample of general practitioners using a single medical record system vs EHR data aggregated from multiple systems) and that Peng et al were looking to align estimates with the national Health Survey for England. In contrast, the study by Teixeira et al found that a combination of diagnosis codes, blood pressure values, and medication data performed well, noting that the use of multiple categories of information improved performance (7). The MENDS-selected e-phenotype — all available criteria for case identification — aligned with clinical reasoning as well as expected levels of hypertension prevalence in the context of available comparison sources.

It is worth noting that a gold standard does not exist for comparing EHR-based estimates of hypertension. BRFSS and NHANES, 2 well-known public health surveillance data sources, differ considerably from each other and from MENDS, which creates challenges in making comparisons. NHANES uses measured blood pressure, similar to MENDS, but data are available only at the national level. BRFSS, whose data are available at the state level, is based on self-reporting of being told one has high blood pressure and does not capture data on those who are undiagnosed. We placed MENDS estimates in the context of these available sources (Table 3). The MENDS 2019 age-adjusted hypertension estimate (41.0%) is between NHANES comparable year’s age-adjusted estimates using a threshold of 130/80 mm Hg (45.4%) (25) and a threshold of 140/90 mm Hg (32.9%) (26). However, the BRFSS national estimate (32.3%) shows that Louisiana (37.1%) trends higher than the national average (18).

In determining how to handle multiple blood pressures captured in a single day, we found little difference in estimates when we used different methods. Thus, we opted to implement the NHANES strategy of averaging all readings from a single day, given NHANES also reflects a public health surveillance system. This approach also makes maximal use of all available information, unlike the eCQM strategy of using only a patient’s lowest values. Strategies to improve blood pressure control, such as the practice of self-measured blood pressure monitoring, can generate additional blood pressure values that could be integrated into EHRs for use in patient care and treatment (29–31). When even more blood pressure values become widely available, additional methods may be needed to efficiently process and use these data for EHR-based surveillance.

### Limitations

This analysis has several limitations. First, included data were restricted to a single state from a single MENDS data contributor to allow for a state-level comparison with BRFSS. This data contributor may not have captured data on all health care encounters and records for each patient, which could have led to underestimates of hypertension prevalence. Second, data from a single data contributor may not be comparable in terms of patient population and data structure with other organizations that aggregate EHR data, so observed hypertension estimates (and relative effects of each decision point in the e-phenotype selection process) may not be generalizable to other health care data sources. Third, existing literature lacked guidance on how to develop a hypertension e-phenotype that would be appropriate for a national EHR-based surveillance system, and therefore, we had a limited evidence base to guide decisions. Thus, we used clinician input, face validity, and comparison with BRFSS estimates for e-phenotype selection. Our decisions were based on clinical reasoning and the observed effect on prevalence estimates (and their comparison with BRFSS estimates) rather than clinical record medical review or direct clinical measures in a sample of individual patients. We did not compare the effect of different analytic choices in an independent EHR-based surveillance system to see whether the effects on hypertension prevalence estimates were similar to each other in magnitude and comparability with BRFSS. Fourth, the misclassification of cases is also possible because some antihypertensive medications can be used to treat conditions other than hypertension. Our review of the top 5 diagnosis codes for patients who used antihypertensive medication and lacked a hypertension diagnosis or high blood pressure measurement were 1) encounter for immunization, 2) other long-term (current) drug therapy, 3) mixed hyperlipidemia, 4) gastroesophageal reflux disease without esophagitis, and 5) hyperlipidemia, unspecified.

### Conclusion

Our analysis helps to address an important knowledge gap by optimizing a surveillance definition of hypertension using EHR data and describing the analytic decisions used in refining the hypertension e-phenotype and their effect on estimates of hypertension prevalence and control. As a result of this analysis, the MENDS team has updated its code and deployed the updated code to its data contributors. Beyond the MENDS project, the selected hypertension e-phenotype may inform clinical and public health efforts to conduct surveillance of hypertension prevalence and control in other data systems and populations. This work underscores the large effect of different analytic decisions to define both numerators and denominators on EHR-based estimates of chronic disease prevalence and control. Our work highlights the importance of transparency in analytic decisions, the potential value of sensitivity analyses to quantify the effect of different analytic decisions, and the importance of future comparative analyses of these methods across different jurisdictions. Future work could validate EHR-based estimates of chronic diseases from independent surveillance systems, while being attentive to the strengths and limitations of these comparators.
