# Erratum, Volume 20, September 14 Release

**DOI:** 10.5888/pcd20.230026e

**Published:** 2023-10-05

In the article “Development of a Hypertension Electronic Phenotype for Chronic Disease Surveillance in Electronic Health Records: Key Analytic Decisions and Their Effects,” by Hohman et al, the first sentence in the Methods section of the Abstract was updated to correct the number of patients. The corrected sentence is, “We used MENDS data from 1,671,544 patients in Louisiana to examine the effect of different analytic decisions on estimates of hypertension prevalence.”

The article was corrected on September 19, 2023, and appears at https://www.cdc.gov/pcd/issues/2023/23_0026.htm. We regret any confusion this error may have caused.

